# Ethylene and 1-Aminocyclopropane-1-carboxylate (ACC) in Plant–Bacterial Interactions

**DOI:** 10.3389/fpls.2018.00114

**Published:** 2018-02-22

**Authors:** Francisco X. Nascimento, Márcio J. Rossi, Bernard R. Glick

**Affiliations:** ^1^Departamento de Microbiologia, Universidade Federal de Santa Catarina, Florianópolis, Brazil; ^2^Department of Biology, University of Waterloo, Waterloo, ON, Canada

**Keywords:** ethylene, 1-aminocyclopropane-1-carboxylate, bacteria, microbiome, plant growth

## Abstract

Ethylene and its precursor 1-aminocyclopropane-1-carboxylate (ACC) actively participate in plant developmental, defense and symbiotic programs. In this sense, ethylene and ACC play a central role in the regulation of bacterial colonization (rhizospheric, endophytic, and phyllospheric) by the modulation of plant immune responses and symbiotic programs, as well as by modulating several developmental processes, such as root elongation. Plant-associated bacterial communities impact plant growth and development, both negatively (pathogens) and positively (plant-growth promoting and symbiotic bacteria). Some members of the plant-associated bacterial community possess the ability to modulate plant ACC and ethylene levels and, subsequently, modify plant defense responses, symbiotic programs and overall plant development. In this work, we review and discuss the role of ethylene and ACC in several aspects of plant-bacterial interactions. Understanding the impact of ethylene and ACC in both the plant host and its associated bacterial community is key to the development of new strategies aimed at increased plant growth and protection.

## Introduction

Plants play a vital role in the Earth’s ecosystems. Their ability to photosynthesize, transforming light energy into chemical energy (in the form of sugars and other organic compounds), provides the energy (either directly or indirectly) necessary for nearly all lifeforms. Amongst these, bacteria possess a privileged relationship with plants that results from tens of millions of years of co-evolution in the Earth’s soils. A huge amount (from 15% up to 70%) of a plant’s photosynthetically fixed carbon is released into the rhizosphere (the portion of the soil surrounding the roots) (Neumann and Rohmeld, 2001). The bacteria present in the rhizosphere bind to external root tissues and use compounds exuded by plants as energy sources (Philippot et al., 2013). Some bacteria, termed endophytes, not only thrive in the rhizosphere, but can also enter and colonize internal plant tissues (Hardoim et al., 2015). In addition, phyllospheric bacteria colonize aerial plant tissues (e.g., leaf) (Vorholt, 2012). Altogether, rhizospheric, endophytic, and phyllospheric bacteria constitute the plant-associated bacterial community, which plays a vital role in plant growth and development. Yet, members of the plant-associated bacterial community may influence plant growth and development in different and sometimes opposing ways. Plant pathogens negatively affect plant growth and development by deleterious and parasitic actions (e.g., production of toxins and enzymes that degrade plant tissues). On the other hand, beneficial bacteria form mutualistic and symbiotic relationships with the plant host (e.g., rhizobia and leguminous plants), and promote plant growth by enhancing plant mineral uptake, nitrogen fixation, production of plant-growth promoting compounds, degradation of compounds that negatively impact plant growth (e.g., xenobiotics), and providing protection from pathogens (Glick, 2014; Santoyo et al., 2016).

Plants have developed an “immune system” composed of a series of intricate and complex mechanisms that ultimately limit and control its associated bacterial communities (Jones and Dangl, 2006). In addition, leguminous plants tightly control the symbiotic nodulation process by a mechanism termed auto-regulation of nodulation (Ferguson et al., 2010).

Plant hormones actively participate in plant developmental, defense and symbiotic programs. In the center of these processes lies ethylene (ET), a gaseous plant hormone, readily diffusible in plant tissues, that exerts its effects even in very low concentrations. ET not only regulates several aspects of plant growth (Van de Poel et al., 2015), but also participates in defense and symbiotic programs induced by bacteria (Desbrosses and Stougaard, 2011; Guinel, 2015), consequently impacting bacteriome assembly. Moreover, several reports point to the role of the direct ET precursor, 1-aminocyclopropane-1-carboxylate (ACC), in regulating plant developmental (Yoon and Kieber, 2013; Vanderstraeten and Van Der Straeten, 2017) and defense responses (Tsang et al., 2011).

As a consequence of the key role of ET and ACC, many bacteria that are closely associated with plants possess sophisticated mechanisms to sense and modulate ET and ACC levels within plant tissues and in the rhizosphere. Although many of these mechanisms are known and their effects in plant growth are documented, not much is understood about their prevalence in bacterial communities, their impact on the plant microbiome and their effect on overall plant growth.

Here, the role of ET and ACC in plant-bacterial interactions is reviewed and discussed. The impact of ET and ACC in plant development, defense and symbiotic programs, as well as, the bacterial strategies used to modulate plant ACC and ET concentrations are described in detail. Ultimately, understanding the impact of ET and ACC in plants and their associated bacteria is key to the development of new technologies aiming to maximize plant growth and protection. A list of the abbreviations used in this study is presented in the Supplementary Material.

## Plant ACC and ET Biosynthesis and Signaling Mechanisms

**FIGURE 1:**
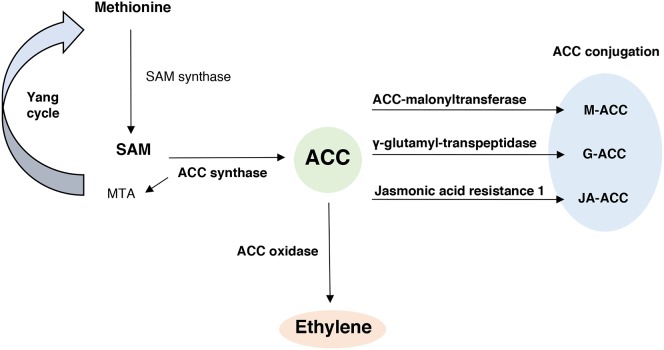
The ethylene biosynthetic pathway and ACC conjugation process. In this pathway methionine is converted to *S*-adenosyl methionine (SAM) by the enzyme SAM synthase. SAM is converted to ACC (1-aminocyclopropane-1-carboxylate) and 5-methylthioadenosine (MTA), by the enzyme ACC synthase. MTA is reconverted to methionine by a series of biochemical reactions, described as the Yang cycle. The enzyme ACC oxidase catalyzes the conversion of ACC to ET. In addition, ACC can be conjugated to M-ACC (Malonyl-ACC), G-ACC (γ-glutamyl-ACC) or JA-ACC (Jasmonoyl-ACC) by the action of the enzymes ACC-malonyl transferase, γ-glutamyl-transpeptidase and Jasmonic acid resistance 1, respectively.

ET biosynthesis occurs in all higher plants via a methionine-dependent pathway (**Figure 1**), in which methionine is converted to *S*-adenosyl methionine (SAM) by the enzyme SAM synthase. SAM is then converted to ACC, the direct precursor of ET, and 5-methylthioadenosine (MTA), by the enzyme ACC synthase (ACS). MTA is reconverted to methionine by a series of biochemical reactions, described as the Yang cycle (Yang and Hoffman, 1984), which replenish the pool of methionine available. Finally, the enzyme ACC oxidase (ACO) converts ACC into ET, HCN and CO_2._

ACC can also be conjugated to form malonyl-ACC (M-ACC), γ-glutamyl-ACC (G-ACC) and jasmonoyl-ACC (JA-ACC) by the action of the ACC-*N*-malonyl transferase (AMT), γ-glutamyl-transpeptidase (GGT) and jasmonic acid resistance 1 (JAR1) enzymes, respectively (**Figure 1**). The conjugation of ACC can also regulate future ACC and ET biosynthesis (Van de Poel and Van Der Straeten, 2014).

### ACC Synthase and ACC

The ET precursor, ACC, is a non-proteinogenic α-amino acid synthesized from SAM by ACS and its production involves transcriptional, post-transcriptional, translational and post-translational regulation (Lee et al., 2017). In all higher plants that have been examined to date, the ACS enzyme is encoded by a multigene family, leading to the production of several isoforms that have specific roles in different plant cells, tissues and developmental processes (Yamagami et al., 2003; Tsuchisaka and Theologis, 2004; Tsuchisaka et al., 2009). Furthermore, ACS can form active heterodimers that may influence their biological activity, regulation and coordination of ACC and ET production (Tsuchisaka and Theologis, 2004; Tsuchisaka et al., 2009; Lee et al., 2017).

The expression of ACS isoforms is controlled at the transcriptional level, with several internal and external cues modulating the transcription of specific ACS genes. Developmental stages, stress conditions and the presence of phytohormones, such as auxin and ET itself, are amongst the main inducers of transcriptional changes in ACS genes (Wang et al., 2005; Vanderstraeten and Van Der Straeten, 2017).

Numerous transcription factors can bind to ACS gene promoters (Lin et al., 2009; Matarasso et al., 2005; Ito et al., 2008; Zhang et al., 2009; Datta et al., 2015). For example, Li et al. (2012) demonstrated that the mitogen-activated protein kinases, MPK6 and MPK3, regulate the expression of *Arabidopsis* ACS2 and ACS6 genes via the WRKY33 transcription factor that binds to the W-boxes (TTGACT/C) in the promoters of ACS2 and ACS6 genes *in vivo*.

Based on their biochemical and structural properties, namely the presence of phosphorylation sites in the C-termini, ACS can be divided in three different groups (Chae and Kieber, 2005). Group I ACS (ACS2 and ACS6) contain phosphorylation sites for both MPKs and CDPKs (calcium-dependent protein kinases). Group II ACS (ACS4, ACS5, ACS8, ACS9, and ACS11) are uniquely phosphorylated by CDPKs, and group III ACS (ACS7) does not contain any phosphorylation sites. These phosphorylation sites have an important role in the increased activation or deactivation of some ACS isoforms, in which kinases, phosphatases, and the ubiquitin-proteasome system play a pivotal role (reviewed by Xu and Zhang, 2014).

### ACC as an Internal Plant-Signaling Molecule

Importantly, Tsuchisaka et al. (2009) demonstrated that the disruption of all *Arabidopsis* ACS isoforms leads to lethality, further indicating the indispensable role of ACS and ACC in plant growth and development. Furthermore, the authors suggest that ACC itself, independently of ET, may play a role as a signaling molecule that controls plant growth and development. Results obtained by Xu et al. (2008) and Tsang et al. (2011) indicate that ACC takes part of a rapid signaling mechanism controlling root cell elongation that is independent of ET signaling. The *Arabidopsis fei1 fei2* mutant plants, defective in the production of the Leucine-Rich Repeat Receptor Kinases (LRRK) FEI1 and FEI2, displayed a severe defect in anisotropic root growth due to a reduced cellulose microfiber content in the cell wall at the root tip. Application of ET biosynthesis inhibitors reversed the *fei1 fei2* phenotype while ET signaling inhibitors did not. Moreover, the ET insensitive mutants *etr1* and *ein2* did not suppress the *fei1 fei2* phenotype. Interestingly, the FEI proteins interacted directly with ACS5 and ACS9 (Xu et al., 2008). Similarly, ET biosynthesis inhibitors reduced the rapid effect of cell wall stress damage induced by isoxaben (a cellulose biosynthesis inhibitor), while the ET signaling mutants *ein3 eil1* presented similar root elongation inhibition as the wild-type plant (Tsang et al., 2011). Both the application of isoxaben and ACC led to a rapid reduction in root epidermal cell elongation in both wild-type and *ein3 eil1* mutants, however, ET signaling components were required for long-term growth responses (Tsang et al., 2011).

ACC and some of its conjugated forms can be readily transported (in a matter of minutes) within the tissues of various plants, via phloem and xylem, further indicating their importance as signaling molecules. For example, ACC can be transported from roots to leaves (long distance transport) and, in a more localized fashion, from cell to cell (short distance transport). Moreover, different cells or organs have different ACS and ACO expression profiles, and ACC may be synthesized in one cell or organ and converted to ET in another cell or organ (reviewed by Vanderstraeten and Van Der Straeten, 2017).

Curiously, the fact that ACC conjugates with other phytohormones such as jasmonic acid, a hormone closely linked to plant defense (Wasternack and Hause, 2013) suggests a role for ACC in phytohormone crosstalk and a possible effect in mediating some plant defense responses.

### ACC as an External Signaling Molecule

The use of exogenous ACC as a mean to study ET effects on plant growth and development is a common practice amongst plant physiologists. Application of ACC to the plant growth medium often leads to the plant triple response phenotype (Merchante and Stepanova, 2017). This is possibly due to the presence of a mechanism responsible for ACC transport across the plant cell wall and membrane, leading to ACC uptake (Shin et al., 2015). Importantly, ACC can be exuded by seeds and roots (Finlayson et al., 1991; Penrose et al., 2001; Penrose and Glick, 2001), indicating the existence of a mechanism responsible for ACC exudation. Intriguingly, there is no genetic information about this mechanism. Under stressful conditions plants can produce highly elevated levels of ACC that subsequently increase ET concentrations (stress ET), leading to an inhibition of plant growth and development (Abeles et al., 1992). Hence, releasing ACC to the rhizosphere may be a useful strategy to decrease the negative effects of ACC and ET accumulation under stress conditions. Moreover, since ACC is readily diffused in water it can easily be taken up by bacteria or nearby root systems; thus, the released ACC may act as a signal to recruit beneficial bacteria and/or signal nearby plants.

### ACC Oxidase and ET

The plant produced ACC is converted to ET by the action of the enzyme ACO, which is also encoded by a multigene family in higher plants (Dorling and McManus, 2012; Ruduś et al., 2013). In *Arabidopsis*, a total of five ACO genes are found, however only ACO2 and ACO4 have been studied in detail (Gómez-Lim et al., 1993; Raz and Ecker, 1999; Ramonell et al., 2002; Raghavan et al., 2006; Linkies et al., 2009). These studies revealed that ACO is induced in several plant tissues by numerous treatments, such as, wounding, ethrel (a liquid compound that is transformed into ET), Fe^2+^, 2,4-dichlorophenoxyacetic acid and ACC. Likewise, several studies have demonstrated the induction of ACO gene expression by biotic and abiotic stresses, phytohormone treatments (including ET) and developmental and ontological cues in other plant species (reviewed by Dorling and McManus, 2012).

Like ACS, the ACO enzyme isoforms are expressed under tissue specific conditions and different translational regulation mechanisms control their production (Dorling and McManus, 2012). In addition, ACO expression can also be affected by post-transcriptional and post-translational regulatory mechanisms (Datta et al., 2015).

### ET Signaling

**FIGURE 2:**
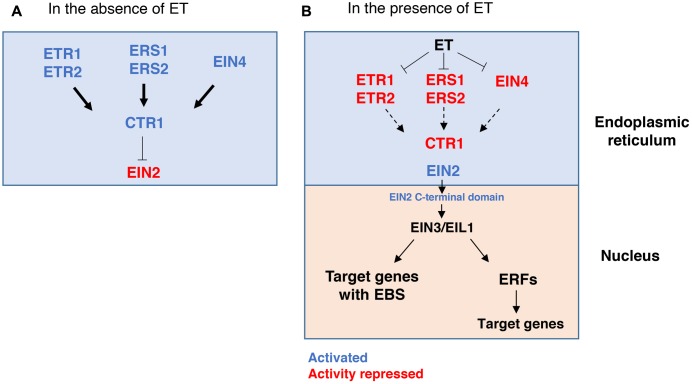
Overview of the ethylene signaling pathway in *Arabidopsis thaliana.* **(A)** In the absence of ET, and, in the endoplasmic reticulum, ET receptors activate the CTR1 kinase that, consequently, phosphorylates EIN2, which becomes inactive and does not induce the ET response. **(B)** In the presence of ET, the ET receptors bind to ET and lose their CTR1-inducing activity, which in turn leads to a diminished activation of CTR1 and, consequently, the activation of EIN2. In this way, the C terminal domain of EIN2 is cleaved and migrates to the nucleus where it induces the expression of EIN3/EIL1 transcription factors, and, subsequently, ERFs and ET-responsive genes containing EBS (EIN3-binding sequences) in their promoter regions. Arrows indicate activation, and T-bars indicate repression of the pathway. Thick arrows represent a strong activation of CTR1 by the ET receptors; Dashed arrows denote less signaling activation of CTR1 upon ET binding to the receptors.

Plants possess an intricate mechanism regulating ET perception and consequent ET-induced responses (**Figure 2**) (reviewed in detail by Ju and Chang, 2015). In *Arabidopsis*, ET is perceived by a five-member family of ET receptors, namely Ethylene Response 1 (ETR1), ETR2, Ethylene Response Sensor 1 (ERS1), ERS2 and Ethylene Insensitive 4 (EIN4), that are located in the plant cell endoplasmic reticulum (ER). These receptors act as negative regulators of the ET signaling pathway. When ET is not present, the receptors activate a Ser/Thr kinase named Constitutive Triple Response 1 (CTR1) that suppresses the ET response by phosphorylating Ethylene Insensitive 2 (EIN2), an ER-bound protein. EIN2 is in an inactive state when it is phosphorylated by CTR1 (**Figure 2A**). On the other hand, when ET binds to the receptors it leads to the inactivation of CTR1 and as a result, EIN2 is dephosphorylated and, consequently, its C-terminal domain is released to migrate to the nucleus. There, EIN2 can, directly or indirectly, activate the transcription factors EIN3 and Ethylene Insensitive-Like Protein 1 (EIL1) that, subsequently, bind to the EIN3-binding sequence (EBS) element in the promoter region of various target genes, thus modulating their expression (**Figure 2B**). Some of these are the ETHYLENE RESPONSE FACTORS (ERFs) genes that further modulate the expression of a wide range of other genetic elements, including those involved in the production of other phytohormones (reviewed in detail by Müller and Munneì-Bosch, 2015).

Some additional reports have shown the existence of several regulators of the ET signaling mechanism (reviewed by Wen et al., 2015), which further impact ET-induced responses.

## Old Foes: ET and ACC act as Inhibitors of Bacterial Colonization and the Nodulation Process

### Pathogens

Studies using mutants impaired in ET biosynthesis and signaling demonstrated a direct role for ACC and ET in plant defense against some bacterial pathogens. *Arabidopsis ein2* mutants presented an increased susceptibility to *Erwinia carotovora* subsp. *carotovora* (now reclassified as *Pectobacterium*) infection as the number of viable bacteria was 7-10 times higher in the mutant than in the wild-type plants (Norman-Setterblad et al., 2000). Recently, Guan et al. (2015) showed that *Arabidopsis acs* mutants (that were deficient in the production of ACC) presented a higher susceptibility to *Pseudomonas syringae* infection. The authors also demonstrated that plants with reduced ACC production were colonized to a greater extent by *P. syringae*.

### Rhizobial Symbionts

Rhizobia can form a symbiotic relationship with leguminous plants by inducing the formation of nodules where the rhizobial nitrogen fixation process occurs. Upon the perception of plant flavonoids, rhizobia produce lipochitooligosaccharides, termed nodulation (Nod) factors (NFs) that ultimately induce the plant symbiotic response and the development of nodules. In order to colonize the plant-produced nodule, rhizobia enter the plant root hair cells, and consequently reach the nodule via infection threads, a tubular structure resulting from the invagination of the plant cell membrane. Once in the nodule, rhizobia differentiate into a specialized symbiotic organelle-like form, termed a bacteroid, which is now able to start the nitrogen fixation process, thus, providing nitrogen to the plant host (reviewed by Gage, 2004).

Generally, ET and ACC act as inhibitors of the nodulation process initiated by rhizobial symbionts (reviewed by Guinel, 2015). Several studies revealed that ET and ACC are involved in several phases of the symbiosis process, including, the initial response to bacterial NFs, NF signal transduction, infection thread formation, nodule development, senescence, and abscission (Penmetsa and Cook, 1997; Heidstra et al., 1997; Oldroyd et al., 2001; Prayitno et al., 2006; Larrainzar et al., 2015; Guinel, 2015). For example, Penmetsa and Cook (1997) showed that the *Medicago truncatula* sickle (*skl*) mutant, insensitive to ET, formed an increased number of nodules. The *skl* mutant is defective in a gene homologous to the *Arabidopsis* EIN2 gene (Penmetsa et al., 2008). The silencing of two *Lotus japonicus* EIN2 homologous genes also resulted in increased nodule formation (Miyata et al., 2013). On the other hand, application of exogenous ET or ACC greatly reduces nodule formation in several leguminous plants (Okazaki et al., 2004).

### Bacterial Endophytes

A bacterium can be considered an endophyte when isolated from internal and asymptomatic plant tissues. This definition encompasses, neutral, commensal and/or beneficial, dormant saprobes and latent bacterial pathogens (reviewed by Compant et al., 2016). In addition, two distinct classes of endophytes can be considered: obligatory endophytes, which spend its entire life cycle inside the plant host; and, non-obligatory endophytes, which spend part of their lifetime inside a plant host but may survive in different environments, such as the rhizosphere (which is the case of most studied bacterial endophytes). In this sense, it has been demonstrated that ET and ACC regulate the interaction between plants and non-obligatory bacterial endophytes. For example, Iniguez et al. (2005) demonstrated that ET acts as an inhibitor of the endophytic colonization process by the nitrogen-fixing endophytic bacterium *Klebsiella pneumoniae* 342, which presented a hyper-colonization phenotype when inoculated in the *skl* mutant of *M. truncatula*. Furthermore, the addition of ACC to the growth medium greatly reduced the *K. pneumoniae* 342 and *Salmonella typhimurium* 14028 endophytic colonization abilities in wild-type alfalfa and wheat seedlings. Conversely, addition of the ET perception inhibitor, 1-methylcyclopropane, resulted in increased endophytic colonization by these strains, in wild-type plants. Curiously, the ET-mediated inhibition of endophytic colonization was decreased in *S. typhimurium* 14028 mutant strains lacking flagellin *fliC* and *fljB* genes, and, type III secretion system genes *spaS* and *sipB*, compared to the wild-type strains, suggesting that ET responses are dependent on host perception of bacterial microbe-associated molecular patterns (MAMPs) and effectors.

### Phyllospheric Bacteria

The *Arabidopsis ein2* mutant displayed a modified phyllospheric bacterial community when compared to the wild-type plant, supporting the role of ET in controlling the phyllosphere microbiota (Bodenhausen et al., 2014). A higher bacterial abundance, as measured by relative 16S rRNA gene copy number, was observed in the *ein2* mutant. Moreover, *Variovorax* strains were more abundant in the phyllosphere of *ein2* mutant plants compared to wild-type plants (Bodenhausen et al., 2014). Together with the results obtained for leaf-associated pathogens, such as *P. syringae*, these results support the effect of ET and its signaling mechanism as a general inhibitor of leaf bacterial colonization.

## Zoom in: ET and ACC Regulate the Plant Immune and Symbiotic Responses

### MAMPs and DAMPs: Pattern Triggered Immunity

The first level of plant inducible defense mechanisms is activated upon recognition of bacterial colonizers and their MAMPs, like flagellin (FLG), elongation factor Tu (EF-Tu), peptidoglycan (PGN), lipopolysaccharides (LPS) and necrosis and ethylene-inducing peptide 1 (Nep1)-like proteins (NLPs) (Newman et al., 2013; Böhm et al., 2014). Additionally, primary plant defenses are also activated in response to damage-associated molecular patterns (DAMPs) (Yamaguchi and Huffaker, 2011) that result from the direct action of invading bacteria (e.g., production of extracellular enzymes and peptides) or from plants’ endogenous peptides and other compounds that may be released following bacterial colonization.

MAMPs and DAMPs are recognized by plant pattern recognition receptors (PRR), subsequently leading to the activation of the pattern-triggered immunity (PTI) response (Jones and Dangl, 2006; Yamaguchi et al., 2010) in which, ion fluxes, intricate MPK signaling cascades, ACC and ET biosynthesis, reactive oxygen species (ROS) production, hydroxyproline-rich glycoproteins (HPRGs), cell-wall strengthening, callose deposition, and gene transcriptional and translational reprogramming take part (Felix et al., 1999; Asai et al., 2002; Zipfel et al., 2004; Boller and Felix, 2009; Luna et al., 2011; Xu et al., 2017).

**FIGURE 3:**
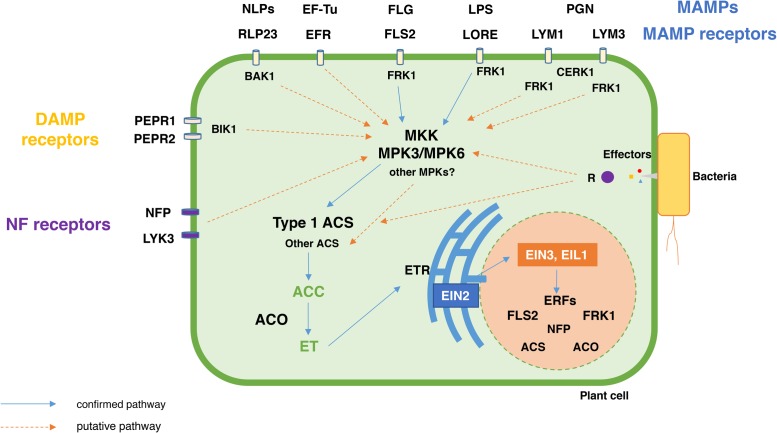
Schematic representation of the MAMPs, DAMPs, NF, and effectors-mediated activation of the ACC and ET biosynthesis and signaling pathways. MAMPs, DAMPs and NFs bind to their cognate receptors present in the plant cell outer membrane and, consequently, initiate the respective signal transduction pathways that lead to the production of ACC and ET. Since most MAMPs, DAMPs and NFs are known to activate MKK-MPK signaling cascades, a MAMP, DAMP and NF-triggered MKK-MPK3/6 cascade (based on *Arabidopsis* gene nomenclature) seems to play a central role in the phosphorylation, and, subsequent activation of type 1 ACS (e.g., AtACS2 and AtACS6). Nevertheless, some aspects of the MAMP, DAMP, NF, and effector induced-R protein signal transduction pathways remain elusive. For example, effector induced immunity leads to the production of ACC and ET, however, not much is understood about the role of effectors and R proteins in the activation of the ET biosynthesis and signaling pathways. MAMP, Microbe Associated Molecular Pattern; DAMP, Damage Associated Molecular pattern; FLG, Flagellin; EF-Tu, Elongation factor-Tu; LPS, Lipopolysaccharide; PGN, Peptidoglycan; NLPs, necrosis and ethylene-inducing peptides; NF, Nodulation factors; FLS2, flagellin receptor; EFR, elongation factor-Tu receptor; RLP23, necrosis and ethylene- inducing peptides receptor; LORE, lipopolysaccharide receptor; LYM1 and LYM3, peptidoglycan receptors; NFP and LYK3, nodulation factor receptors; PEPR1-2, Pep1 receptor. R, resistance protein involved in effector recognition; CERK1, LysM receptor kinase; FRK1 -Flg22-Induced Receptor-Like Kinase 1; BAK1, Brassinosteroid insensitive 1 (BRI1)-associated kinase; BIK1, Botrytis-Induced Kinase 1; MKK, mitogen-activated protein kinase kinase; MPK, mitogen-activated protein kinase. ACS, ACC synthase; ACO, ACC oxidase; ERF, Ethylene Response Factor.

ET plays an important role in PTI, and in some cases, it acts both upstream and downstream of the PTI response (**Figure 3**). The accumulation of the LRRK FLS2 (Flagellin Sensitive 2), the receptor for the bacterial flagellin or its active epitope Flg22, is reduced in ET-insensitive *etr1* and *ein2* mutants, indicating a requirement of ET signaling for *FLS2* increased expression and consequent Flg22-induced responses (Mersmann et al., 2010; Boutrot et al., 2010; Tintor et al., 2013). Moreover, Boutrot et al. (2010) observed that FLS2 is positively regulated by EIN3 and EIN3-like transcription factors. The application of exogenous ACC also leads to an increased expression of *FLS2* (Tintor et al., 2013). Similarly, the FRK1 (Flg22-INDUCED RECEPTOR-LIKE KINASE 1) gene, which is activated in response to Flg22, and, acts downstream of FLS2 (Asai et al., 2002), is influenced by ET; transcript levels of FRK1 are reduced in *ein2-5* mutants after Flg22 treatment (Boutrot et al., 2010).

Importantly, Flg22 treatment induces the activation of several defense related genes trough a MPK signaling cascade (Asai et al., 2002). Moreover, Flg22 induced the increased expression of MPK3 and MPK6, but no other MPK isoforms (Asai et al., 2002). This is consistent with previous studies showing MPK6 activation following Flg22 treatment (Nühse et al., 2000). The stress-responsive MPK3 and MPK6 phosphorylate the ACS2 and ACS6 isoforms, thus, leading to an increased level of ACC and, consequently, ET production (Liu and Zhang, 2004; Li et al., 2012).

An increase in ET production was also observed in response to EF-Tu (Kunze et al., 2004). *Arabidopsis ein2* mutants present a decreased sensitivity to the EF-Tu epitope, elf18 (Tintor et al., 2013), however, the expression of the EF-Tu Receptor (EFR) is not affected in the *ein2* mutant, suggesting that ET acts downstream of the EFR-dependent responses. Tintor et al. (2013) observed that a dysfunctional ET signaling mechanism causes improper transcriptional reprogramming during EFR-triggered immunity. Recently, Xu et al. (2017) demonstrated that genes involved in the ET response were amongst the genes with translational efficiency changes in plants challenged with elf18. *Arabidopsis ein4-1*, *erf7*, and *eicbp.b* (ETHYLENE INDUCED CALMODULIN BINDING PROTEIN) mutants displayed insensitivity to elf18-induced resistance (Xu et al., 2017).

Recently, a leucine-rich repeat receptor protein (LRR-RP) RLP23 has been shown to act as the receptor for NLPs (nlp20) and act together with the SOBIR1 [Suppressor of Brassinosteroid Insensitive 1 (BRI1)-associated kinase (BAK1)-interacting receptor kinase 1], and BAK1 proteins to produce the NLP-induced defense response (Albert et al., 2015). NLPs are abundant in bacteria and can also be considered MAMPs (Böhm et al., 2014; Oome et al., 2014). *Bacillus halodurans* and *B. subtilis* nlp20 peptides trigger ET production in *Arabidopsis* (Böhm et al., 2014).

Peptidoglycan from several Gram-positive and Gram-negative bacterial strains are recognized by plant lysin-motif (LysM) domain proteins, LYM1 and LYM3 (Willmann et al., 2011). Acting downstream, a membrane LysM receptor kinase (CERK1) is also required to induce transcriptional responses induced by PGN. This signal transduction mechanism leads to the activation of FRK1, whose expression has been shown to be regulated by ET, suggesting a role for ET in PGN-induced responses.

Ranf et al. (2015) revealed that a plant lectin S-domain-1 receptor–like kinase, LORE, is responsible for the recognition of bacterial LPS. *Arabidopsis* mutants, *lore-1* and *lore-2*, present a diminished LPS-triggered accumulation of ROS, activation of MPK3 and MPK6 and expression of PTI response genes, such as FRK1 and GST1 (GLUTATHIONE-*S*-TRANSFERASE 1) (both of which are ET regulated). These results suggest that LPS-induced responses may modulate ACS expression in a MPK3-6 dependent manner, as previously observed by Li et al. (2012), and thereby induce the expression of ET-responsive proteins like FRK1.

DAMPs induce ET production and modulate ET responses. Nevertheless, ET itself can induce the production of DAMPs, indicating a role for ET acting upstream and downstream of the DAMP-induced response. Upon wounding, methyl jasmonate or ET application, *Arabidopsis* produces Pep1, a 23-amino acid peptide processed from PROPEP1 (Precursor of Peptide 1), which binds to the Pep1 receptor kinases PEPR1 and PEPR2 (Huffaker et al., 2006; Yamaguchi et al., 2010; Liu et al., 2013). PEPR1 and PEPR2 directly phosphorylate the BOTRYTIS-INDUCED KINASE 1 (BIK1) in response to Pep1 treatment (Liu et al., 2013). *Arabidopsis pepr1/pepr2* and *bik1* mutants present a compromised ET-induced expression of defense genes. Curiously, *pepr1/pepr2* mutants displayed a reduced sensitivity to ET, suggesting a direct effect in the ET signaling pathway (Liu et al., 2013).

Other studies have shown that the application of several bacterial extracellular enzymes that impact plant tissues (e.g., pectate lyase) induce ET production in several plant species (reviewed in Abeles et al., 1992). However, the effect of all of these applications has not been studied in detail.

### Effector-Triggered Immunity

In addition to transmembrane PRR, plants also produce specific defense nucleotide binding and leucine rich repeat domains (NB-LRR) proteins inside the cell (Jones and Dangl, 2006). These plant resistance (R) proteins are involved in the second layer of defense, which is induced upon recognition of specific effectors that are produced by bacteria able to suppress or evade PTI. The R proteins recognize bacterial effectors, thus, initiating effector-triggered immunity (ETI) (Jones and Dangl, 2006). The ETI response is frequently associated with hypersensitive response cell death (HR) (Jones and Dangl, 2006; Dodds and Rathjen, 2010). ET production is closely linked with ETI and HR. Some studies have revealed that following pathogen infection (*P. syringae*), ET is produced in a biphasic pattern in both *Nicotiana tabacum* and *Arabidopsis* plants (Mur et al., 2008, 2009). The first ET peak seems to be related with PTI and it is rapidly induced. The generation of the second ET peak is dependent on ETI, as bacteria deficient in effector delivery (*hrpL* mutants) are not able to induce the second ET peak (Mur et al., 2008). Also, the bacterial *avr* gene and its ETI-inducing activity is closely related to the second ET peak production (Mur et al., 2008, 2009). Guan et al. (2015) showed that the *P. syringae2* (*rps2*) *Arabidopsis* mutant seedlings lacking the R protein and, therefore, unable to sense the *avrRpt2* effector, produced decreased effector stimulated ET levels. On the other hand, Liu et al. (2011) showed that the *Erwinia amylovora*-derived elicitor HrpNEa activates the transcription factor MYB44, which in turn enhances the expression of *EIN2*. Recently, Blüher et al. (2017) demonstrated that *Xanthomonas campestris* pv. *vesicatoria* produces a type III secretion effector, XopH, that possesses phytase activity and modulates the *Nicotiana benthamiana* defense response. The authors observed that XopH induced the expression of *N. benthamiana* ET-responsive genes encoding the pathogenesis-related proteins, PR1b, PR4 and the proteinase inhibitor PI-II. Moreover, the expression of *PR4* and *PI-II* genes were dependent on the ET signaling pathway, as silencing of ET pathway components, such as EIN2, suppressed their upregulation.

Studies using *Arabidopsis* ET-overproducing (ETO) and signaling mutants indicate that ET strongly participates in the HR response (Mur et al., 2009). For instance, *eto2-1* mutants (overproducing ACC and ET) induce an exaggerated HR, while ET insensitive mutants (*ein2-1* and *etr1-1*) present a delayed HR.

### Symbiotic Nod Factor-Triggered Response

Rhizobial NF perception by a leguminous plant leads to the initiation of the symbiotic program, which ultimately results in nodule formation and biological nitrogen fixation (reviewed by Guinel, 2015). Bacterial NFs are perceived by plant NF receptors, such as the lysine motif domain-containing receptor-like kinase 3 (LYK3) and nodulation factor perception (NFP) in *M. truncatula*, and nodulation factor receptor1 (NFR1) and NFR5 in *Lotus japonicus*. The NF receptors are plasma membrane-localized receptor-like kinase and kinase-like (RLK) proteins (Amor et al., 2003; Smit et al., 2007; Moling et al., 2014) containing an intracellular kinase domain and an extracellular region with two or three chitin-binding LysM motifs, which bind to NFs through their chitin backbone (Petutschnig et al., 2010; Broghammer et al., 2012).

The *M. truncatula skl* mutant root transcriptome revealed the important role of ET in the NF-signaling cascade and the overall nodulation process. Larrainzar et al. (2015) observed that the *skl* mutant presented an increased expression of NFP, LYK3 and several members of the LysM kinase family, further indicating that ET impacts NF receptor gene transcription. Furthermore, ET also regulates the transcriptional response that occurs downstream of NF perception, including ACS and ACO expression, as well as other genes involved in the production of other phytohormones (Larrainzar et al., 2015). Several other events occurring after NF perception, such as calcium spiking, root hair deformation, infection thread formation and persistence, and primordium formation at sites opposite phloem poles, have been demonstrated to be affected by ET (Penmetsa and Cook, 1997; Heidstra et al., 1997; Oldroyd et al., 2001; Larrainzar et al., 2015).

Importantly, Larrainzar et al. (2015) also identified the presence of a NF-independent and ET-modulated response in *M. truncatula* plants challenged with rhizobial symbionts. This response likely accounts for the PTI and ETI immune response elicited by rhizobial symbionts MAMPs, DAMPs and effectors.

## An ET and ACC-Regulated Mechanism Controlling Development and Defense? the Root Cell Elongation Example

Plant developmental cues and defense responses are intrinsically related and may act synergistically to limit bacterial proliferation. Even though ET and ACC directly impact the fast and localized plant immune and symbiotic response, ET and ACC are also known for their effects in long-term plant development, especially in the modulation of root growth and development. In this sense, ET and ACC act mainly as negative regulators of the root cell elongation process.

The ACC (or ET)-induced inhibition of root cell elongation is a very fast mechanism (i.e., it occurs within minutes) and is mediated by several other players, such as ROS, HPRGs, plasma membrane H^+^-ATPases and other enzymes involved in cell-wall remodeling (Le et al., 2001; De Cnodder et al., 2005; Staal et al., 2011, Markakis et al., 2012). The crosslinking of HPRGs by ROS and the quick deposition of callose in the apoplast (the main colonization spot of bacterial endophytes) contribute to cell elongation arrest and the general inhibition of root elongation induced by ACC (axenic seedlings) (De Cnodder et al., 2005). In addition, ACC induces apoplastic alkalinization in root cells that leads to a decrease in the activity of cell-wall loosening agents which function in more acidic environments. The alkalinization occurs as a consequence of changes in H^+^ efflux by the modulation of the activity state of plasma membrane H^+^-ATPases (Staal et al., 2011). After a 3-h treatment with ACC, the expression of several genes coding for known cell-wall loosening proteins are down regulated, while genes coding for specific cell wall components together with their cross-linking enzymes (e.g., peroxidases) are upregulated (Markakis et al., 2012).

Application of ACC also leads to an increased synthesis and a modified transport of auxin, which readily impacts root developmental programs (reviewed by Muday et al., 2012). Moreover, auxin and its signaling mechanism are necessary for the ACC and ET-induced root elongation inhibition in *Arabidopsis* (Swarup et al., 2007; Stepanova et al., 2007; Ruzicka et al., 2007; Strader et al., 2010; Staal et al., 2011).

Interestingly, most of the effects identified in ACC and ET-induced responses (single application of ACC or ET) are also observed in the immune responses induced by bacteria and their MAMPs. Plant immune responses rapidly induce the production of ACC and ET, modify ion fluxes and induce growth medium alkalinization due to changes of ion fluxes across the plasma membrane, induce increased ROS production and accumulation (Boller and Felix, 2009) and increase HPRGs in the cell-wall (reviewed by Deepak et al., 2010). The crosslinking of these glycoproteins and the consequent strengthening of the cell wall in response to microbial invaders is dependent on the action of ROS (mainly H_2_O_2_) and peroxidase enzymes (Deepak et al., 2010). Moreover, callose deposition is induced by MAMPs (Luna et al., 2011). The plant immune response usually leads to seedling growth inhibition (Boller and Felix, 2009).

Importantly, Tsang et al. (2011) demonstrated that the application of isoxaben (an inhibitor of cellulose synthesis and general root development) or Flg22, induced root cell elongation arrest in *Arabidopsis*. The application of ET biosynthesis inhibitors reduced the negative effects of both isoxaben and Flg22, indicating a similar mechanism regulating root elongation inhibition induced by these compounds. Tsang et al. (2011) further indicated that an ACC-dependent signaling mechanism, involving auxin and ROS production acting downstream, was responsible for root elongation inhibition.

Altogether, these results are consistent with the existence of a common ET and ACC-mediated mechanism regulating root elongation (and possibly other processes) that can be activated by different elicitors, including MAMPs. The root elongation inhibition process can limit bacterial colonization and degradation of plant compounds since stronger and less elongated cells, containing more antimicrobial compounds (e.g., ROS and callose) and presenting a modified permeability, may be less susceptible to bacterial colonization.

## Counter Attack! Bacterial Modulation of Plant ACC and ET Levels

**FIGURE 4:**
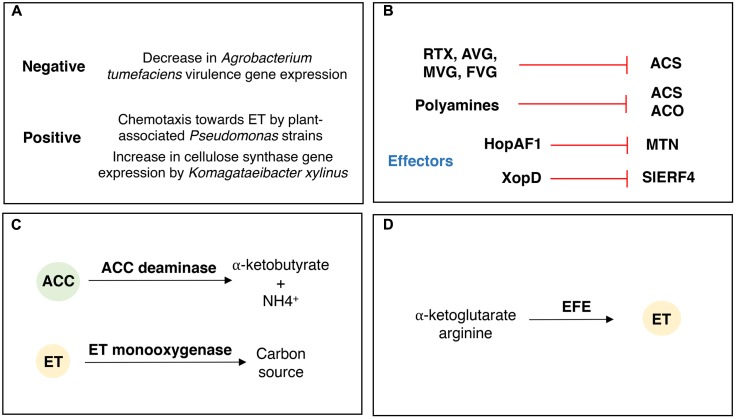
Bacterial mechanisms involved in the responses to ethylene and modulation of plant ACC and ethylene levels. **(A)** Bacterial responses to ET, both positive and negative that relate to the ET effect in the expression of several genes and traits. **(B)** Compounds and effectors impacting plant ACC and ET biosynthesis and signaling. RTX, Rhizobitoxine; AVG, aminoethoxyvinylglycine; MVG, methoxyvinylglycine; FVG, 4-formylaminooxyvinylglycine; MTN, methylthioadenosine nucleosidase. **(C)** Bacterial degradation of plant ACC and ET. Bacteria presenting ACC deaminase activity catabolize ACC to produce α-ketobutyrate and ammonia. Bacteria producing an ET-monoxygenase and other associated components can use ET as sole carbon source. **(D)** Bacterial ET production. Some bacterial pathogens produce ET by the action of an ET-forming enzyme (EFE) that uses arginine and α-ketoglutarate as substrates.

Bacteria have developed several mechanisms to respond and modulate plant ACC and ET levels (**Figure 4**). These mechanisms are related to bacterial physiologic adaptations upon ET perception (**Figure 4A**), and/or production of compounds and effectors that directly or indirectly impact the production and signaling of ET by the plant (**Figure 4B**), modulation of plant ET responses by degrading ACC or ET (**Figure 4C**) or, alternatively, by producing ET (**Figure 4D**).

### Bacterial Responses to ET

Studies on plant-associated bacteria demonstrated that ET impacts the expression of several bacterial genes involved in plant-bacterial interactions. For example, *Agrobacterium tumefaciens* virulence (*vir*) gene expression is negatively affected by exogenous ET, which leads to a decreased ability of T-DNA transfer, and, consequently, to a reduction of pathogenicity (Nonaka et al., 2008). ET induced the increased expression of the cellulose synthesis operon, as well as the CRP/FNR_Kx_ transcription factor, in the fruit-associated bacterium *Komagataeibacter xylinus*, which in turn may favor external bacterial adhesion, competitiveness and consequent production of plant-growth promoting traits (Augimeri and Strap, 2015). These results indicate that the ET impact in plant-associated bacteria responses may be strain specific and dependent on the bacterial mode of action. *Agrobacterium* is a biotrophic pathogen that colonizes internal plant tissues (mainly roots and shoots) and induces tumors. It is conceivable that upon sensing increased ET levels, *Agrobacterium* modulates its virulence through differential *vir* gene expression in order to subvert the plant defense response mediated by ET. On the other hand, *K. xylinus* is an epiphyte colonizing the external surface of fruits. ET plays an important role as a fruit ripening agent (Liu et al., 2015), so, the ET signal may indicate the ideal timing for fruit colonization by *K. xylinus*, which in turn produces a dense cellulose matrix that increases its adherence to the fruit, provides protection from environmental stresses, and provides a competitive advantage over other microorganisms (Augimeri and Strap, 2015).

A study performed by Kim et al. (2007) revealed that *Pseudomonas aeruginosa* PAO1 and many plant-associated bacteria, including *P. fluorescens*, *P. putida*, and *P. syringae*, can perceive and positively respond to ET. The authors identified the methyl-accepting chemotaxis protein (MCP), TlpQ, as the chemoreceptor responsible for ET responses in *P. aeruginosa* PAO1. Moreover, the *cheR* gene encoding a chemotaxis-specific methyl-transferase is required for strain PAO1 MCP-dependent chemotaxis toward ET (Kim et al., 2007). Homologs of the *tlpQ* gene were also identified in several other plant-associated bacteria.

### Inhibition of Plant ACS by Bacterial-Produced Vinylglycine Analog Compounds

#### Rhizobitoxine (RTX)

Rhizobitoxine is a secreted enol-ether amino acid that acts as an inhibitor of the plant ACS (Yasuta et al., 1999). The genes *rtxA* (encoding a dihydrorhizobitoxine synthase) and *rtxC* (encoding dihydrorhizobitoxine desaturase) are responsible for RTX production in *Bradyrhizobium* (Yasuta et al., 2001). Knowledge of the role of RTX in plant-bacterial interactions resulted mainly from studies of the *Bradyrhizobium*-legume symbiosis. *B. elkanii* RTX mutant strains, unable to produce RTX and, consequently, decrease plant ET levels, have decreased nodulation abilities and competitiveness in several plant hosts. Duodu et al. (1999) showed that *B. elkanii* RTX mutant strains formed fewer mature nodules than the wild-type strain in *Vigna radiata*; however, the nodulation profile of the mutant strains could be partially restored by the addition of ET biosynthesis inhibitors. Elimination of RTX production in *B. elkanii* led to increased ET production by *Macroptilium atropurpureum* and a decreased nodulation phenotype (Yuhashi et al., 2000). Similar results were obtained by Parker and Peters (2001) who showed that *Amphicarpaea edgeworthii* plants inoculated with *B. elkanii* RTX-deficient mutants RX17E and RX18E developed fewer nodules than plants inoculated with the wild-type *B. elkanii* USDA 61. Interestingly, Ratcliff and Denison (2009) demonstrated that the RTX-producing *B. elkanii* increased the accumulation (by 47%) of the storage lipid poly-3-hydroxybutyrate (PHB) in root nodules of *M. atropurpureum*, compared to the *B. elkanii* RTX non-producing mutant. The synthesis of PHB supports the later reproduction of rhizobia (Ratcliff et al., 2008), suggesting that RTX-producing bacteria modulate ET levels to decrease plant sanctions against accumulation of carbon compounds at the expense of N_2_ fixation.

RTX-producing bacteria can also induce disease symptoms in some plants. For example, *B. elkanii* causes foliar chlorosis in some soybean cultivars (*Glycine max*) and this effect is dependent on RTX production (Okazaki et al., 2004). Interestingly, some plant pathogens are also able to produce RTX. Mitchell and Frey (1988) showed that the plant pathogenic *Burkholderia andropogonis* strains produce RTX. *Xanthomonas oryzae* is also known to possess *rtx* genes (Sugawara et al., 2006) but its activity has never been described.

#### Aminoethoxyvinylglycine (AVG)

Aminoethoxyvinylglycine is a powerful inhibitor of the ACS enzyme (Icekson and Apelbaum, 1983), and it has been used in many studies regarding the role of ET in plant physiology, as well as in several agricultural applications, such as, harvesting and fruit ripening delay. AVG is an unsaturated enol ether amino acid produced by *Streptomyces* sp. NRRL-5331 in fermentation broth (Pruess et al., 1974), however, not much is understood about the genetic elements involved in AVG synthesis by strain NRRL-5331, nor the biological significance of its possible interaction with a plant. Most of the studies performed with AVG resulted from the knowledge previously obtained by studying RTX.

#### Methoxyvinylglycine (MVG) and Formylaminooxyvinylglycine (FVG)

*Pseudomonas aeruginosa* strains produce another vinylglycine analog, MVG, also known as AMB (L-2-amino-4-methoxy-trans-3-butenoic acid) (Sahm et al., 1973), through the expression of the *ambABCDE* gene cluster (Lee et al., 2010). Application of pure MVG decreased apple ET levels (Mattoo et al., 1979). Lee et al. (2013) showed that the expression of *ambABCDE* by the biocontrol strain *P. fluorescens* CHA0 weakly interfered with the germination of several graminaceous seeds. Curiously, some rhizosphere-associated *P. fluorescens* produce the vinylglycine analog, FVG, a germination-arrest factor that has been shown to limit the germination of weedy grasses (McPhail et al., 2010; Okrent et al., 2017b). The biosynthetic cluster involved in FVG production by *P. fluorescens* WH6 has recently been described (Okrent et al., 2017a), yet, not much is understood about the biological significance of FVG in the bacterial interaction with the plant host. ET is a known inducer of seed germination (Corbineau et al., 2014), so it is possible that FVG and MVG inhibit ACS and ET production that arrests the germination of the seeds of some plants, however, this remains to be conclusively proven.

### Direct Decrease of Plant ACC Levels by ACC Deaminase-Producing Bacteria

Bacteria that produce the enzyme ACC deaminase can directly use plant-synthesized ACC as carbon and nitrogen sources, and, at the same time, lower the ACC levels within plant tissues (Glick et al., 1998; Penrose et al., 2001; Belimov et al., 2009).

ACC deaminase is a pyridoxal phosphate (PLP)-dependent multimeric enzyme (homodimer or homotrimer) belonging to the tryptophan synthase beta superfamily, with a subunit molecular mass of approximately 35–42 kDa and it can degrade ACC and several ACC-related substrates (reviewed by Nascimento et al., 2014). The ACC deaminase enzyme is encoded by a single gene, termed *acdS*, which is widespread in plant-associated bacteria, including symbionts like rhizobia, general rhizospheric and endophytic plant-growth-promoting bacteria such as *P. fluorescens-*group species, as well as some plant pathogens such as *P. syringae* or *Ralstonia solanacearum* (Nascimento et al., 2014).

Beneficial ACC deaminase-producing bacteria enhance plant growth and development and also increase plant tolerance to a wide variety of biotic and abiotic stresses by decreasing inhibitory ACC and ET levels (Wang et al., 2000; Grichko and Glick, 2001; Mayak et al., 2004a,b; Belimov et al., 2005; Belimov et al., 2009; Toklikishvili et al., 2010; Nascimento et al., 2013; Gamalero and Glick, 2015; Gamalero et al., 2016).

Studies using bacterial mutants impaired in ACC deaminase production have demonstrated that the expression of ACC deaminase is extremely important for the plant-growth promoting abilities of several plant-associated bacteria, including rhizospheric (Glick et al., 1994; Li et al., 2000; Belimov et al., 2009), endophytic (Sun et al., 2009; Onofre-Lemus et al., 2009; Ali et al., 2014) and symbiotic rhizobial strains (Ma et al., 2003; Uchiumi et al., 2004).

Inoculation of leguminous plants with ACC deaminase-producing rhizobia, inoculated singly or in consortia with free-living ACC deaminase-producing bacteria, leads to an increased nodulation phenotype (reviewed in Nascimento et al., 2016). By decreasing ACC levels these bacteria diminish the inhibitory ET concentrations that affect several phases of the nodulation process (Ma et al., 2003).

ACC deaminase-producing bacteria are known to increase general root development, with special emphasis on root elongation (Glick et al., 1994; Belimov et al., 2009). Bacterial mutants impaired in ACC deaminase-production no longer promote root elongation in several plant species (Glick et al., 1994; Belimov et al., 2009; Onofre-Lemus et al., 2009). This result is consistent with the role of ET and ACC in controlling the root elongation process, as previously discussed.

ACC deaminase production also plays a role in bacterial competitiveness. The *acdS*^-^ mutant of *Mesorhizobium* sp. MAFF303099 presented decreased nodulation and nodule occupancy abilities when compared to its wild-type counterpart (Uchiumi et al., 2004). On the other hand, rhizobial strains expressing an exogenous *acdS* gene exhibited increased nodule occupancy compared to the wild-type strains (Ma et al., 2004; Conforte et al., 2010).

There are many studies regarding the effects of ACC deaminase in plant-growth promoting bacteria, however, not much is understood about its effect on pathogens like *P. syringae* or *R. solanacearum*, that also contain an *acdS* gene. It is conceivable that pathogens may decrease ACC levels to decrease ET-regulated plant defense responses. Alternatively, these bacteria may decrease ACC and ET levels that impact their own gene expression (e.g., *vir* gene expression in *Agrobacterium*). In fact, engineered *Agrobacterium* strains expressing ACC deaminase presented an increased ability to transfer T-DNA to different plant hosts (Hao et al., 2010; Nonaka and Ezura, 2014), however the effect of ACC deaminase in *vir* gene expression was not documented.

### Bacterial Effectors Targeting Plant ET Biosynthesis and Signaling Pathways

The plant pathogen *Xanthomonas euvesicatoria*, produces an effector which modulates the ET response pathway in tomato. The type III secretion effector, XopD, directly targets and desumoylates the tomato ET-responsive transcription factor, SlERF4, to suppress ET production, and, consequently decrease ET-induced plant defenses (Kim et al., 2013). As a result, *X. euvesicatoria* increases its growth and delays symptom development in the host plant.

A type III effector, HopAF1, produced by *P. syringae* and encoding a deamidase-like enzyme, targets *Arabidopsis* methylthioadenosine nucleosidase proteins MTN1 and MTN2, which are involved in the Yang cycle and, consequently, ET production (Washington et al., 2016). HopAF1 inhibits the MAMP-induced increase in ET biosynthesis, leading to an increased bacterial infection. Additionally, several HopAF1 homologs are found in the genomes of other bacterial pathogens, such as *R. solanacearum* or *Acidovorax citrulli*, consistent with the suggestion that effector production targeting ET responses is an important trait in some plant pathogens (Washington et al., 2016).

### Bacterial Polyamines and the Decrease of Plant ET Levels

Polyamines (PAs) are low-molecular-weight aliphatic amines commonly produced by a large number of different organisms (Miller-Fleming et al., 2015). The most abundant PAs include, spermine and spermidine, and their precursor putrescine. Importantly, the application of PAs have been shown to decrease ACC and ET levels in several plant species, apparently by limiting the action of ACS and ACO enzymes (Li et al., 1992, 2013).

A study by Xie et al. (2014) showed that spermidine produced by *B. subtilis* OKB105 inhibited the expression of tobacco ACO1, consequently, reducing the ET content in root cells, and, thereby increasing tobacco root growth. Spermidine production by *B. subtilis* OKB105 is dependent on the *speB* gene encoding agmatinase. Moreover, the *yecA* gene encoding a putative amino acid/polyamine permease, is responsible for spermidine export (Xie et al., 2014).

### Direct Decrease of ET Levels by Soil Bacteria Expressing ET-Monooxygenase

Several Actinobacteria like *Mycobacterium* and *Nocardioides*, which are common soil inhabitants, possess the ability to use ET as a sole carbon source (de Bont and Harder, 1978) by the expression of an ET-monooxygenase (Coleman and Spain, 2003). The genetic elements responsible for bacterial ET degradation have been described in detail (Coleman and Spain, 2003), however, not much is understood about the role of ET-degrading bacteria in plant development and plant-microbe interactions. Thus, more studies are necessary to elaborate the role of bacterial ET degradation in modulating plant growth.

### The Direct Increase in ET Levels by the Production of a Bacterial ET-Forming Enzyme

Pathogens like *R. solanacearum* and *P. syringae* possess the ability to produce ET, independent of a plant host (Freebairn and Buddenhagen, 1964; Nagahama et al., 1991; Weingart et al., 2001). In this case, bacterial ET production is not ACC-dependent, rather, it depends on the action of an ET-Forming Enzyme (EFE) that uses α-ketoglutarate and arginine as substrates (Nagahama et al., 1991). Importantly, Weingart et al. (2001) demonstrated that a *P. syringae* pv. *glycinea efe* mutant presented a decreased pathogenicity. In addition, the expression of a bacterial EFE in transgenic tobacco plants resulted in altered plant development, with plants demonstrating a dwarf morphology. These results suggest that ET synthesis is extremely important for the action of some pathogens. This leads to intriguing questions: If a low level of ET is responsible for increased plant defenses why do some bacterial pathogenic strains produce ET? Moreover, if *P. syringae* possesses several mechanisms aimed at decreasing plant ACC and ET levels (RTX, ACC deaminase, effectors), why do the same bacterial strains sometimes produce ET?

Depending on environmental and internal cues, ET can either positively or negatively regulate stomatal opening in several plant species (Madhavan et al., 1983; Tanaka et al., 2005; Desikan et al., 2006; Arve and Torre, 2015). Hence, under certain conditions, producing ET may lead to increased leaf colonization by *P. syringae* entering open stomata, or, alternatively, to decreased stomata opening that protects endophytic *P. syringae* from external competitors. Since ET also acts as a chemoattractant, it is also possible that ET production may act as a signaling mechanism in *P. syringae*.

Ethylene is a major inducer of plant stress symptoms and these may be important in the later phases of the bacterial infection process. Bacteria such as *P. syringae* are transmitted mainly by soil and water (van Overbeek et al., 2010; Monteil et al., 2016), so it is possible that in the late disease stages some *P. syringae* strains produce ET to increase foliar senescence and abscission aiming for bacterial dispersal.

## Future Directions

### ACC as a Signaling Molecule Affecting Microbiome Assembly?

Importantly, several studies have shown that ACC deaminase-producing bacteria are enriched in the rhizosphere and seeds of stress-grown plants. Timmusk et al. (2011), showed that ACC deaminase-producing bacteria were much more abundant in the rhizosphere of wild barley growing under stressful conditions in comparison to barley grown nearby under non-stressful conditions. This result was obtained even though both sampled environments had similar soil, rock and topology characteristics. Moreover, ACC deaminase-producing bacteria were abundant in plant rhizosphere samples and almost non-existent in bulk soil samples. Similarly, ACC deaminase-producing bacteria were more abundant in all compartments of heavy metal contaminated soils (bulk and *Brassica napus* rhizosphere) than in non-stressed soils (Croes et al., 2013).

Truyens et al. (2013) studied the cultivable endophytic population of seeds from *A. thaliana* exposed to cadmium for several generations (Cd seeds) in comparison with a population isolated from seeds of plants that were never exposed to Cd (control seeds). The authors found that metal tolerance and ACC deaminase activity were predominantly found in strains isolated from Cd seeds, while the production of siderophores, indole-3-acetic acid and organic acids was more prevalent in endophytes isolated from control seeds, further indicating a selection for ACC deaminase-producing bacteria under stress condition that is consistent with the increased ET/ACC levels induced by cadmium and other heavy-metal stresses (Thao et al., 2015).

Altogether, these results indicate that ACC and ET may act as signaling molecules under stress conditions, leading to an increased recruitment of bacteria able to decrease the elevated ACC and ET levels responsible for decreased root growth and increased plant stress. In turn, ACC and ET-modulating bacteria decrease stress ACC and ET levels, relieving the plant from its negative effects in several plant developmental cues (Glick, 2014). Nevertheless, more studies are necessary to understand the mechanism responsible for plant ACC exudation, as well as, bacterial ACC perception, and their consequent role in the plant microbiome assembly.

### Is Plant Production and Sensitivity to ET and ACC Regulating the Plant Microbiome?

Since ET and ACC impact bacterial colonization, their role gains further importance in microbiome assembly (especially under stressful conditions). In this sense, it is conceivable that plants presenting different ET/ACC production and sensitivity abilities may possess different microbiome selection abilities. Although ET and ACC are produced by all higher plants, the timing and extent of ET/ACC production differs between plant species (Abeles et al., 1992; Wheeler et al., 2004). These differences may be explained by the abundance of genetic elements involved in ET/ACC production in various plant species. For instance, *Arabidopsis* contains 12 ACS isoforms in its genome while 6 ACS isoforms are found in *Lotus japonicus* (Desbrosses and Stougaard, 2011). Similarly, different plants possess different ET/ACC sensitivities (e.g., Woltering and Van Doorn, 1988), which is also consistent with the disparate numbers of genetic elements involved in ET perception and signaling in plant genomes (Desbrosses and Stougaard, 2011). For example, *M. truncatula* only possesses one EIN2 homolog (Penmetsa et al., 2008), while *L. japonicus* contains two EIN2 gene homologs in its genome (Miyata et al., 2013).

### What Is the Contribution of ACC and ET-Modulating Bacteria to the Overall Plant Microbiome?

The ACC deaminase-producing bacterial strain *Pseudomonas* sp. UW4, but not its *acdS*^-^ mutant, increased the colonization of the arbuscular mycorrhizal fungus, *Gigaspora margarita* BEG9 in cucumber, leading to synergistic effects on plant growth (Gamalero et al., 2008). Furthermore, several reports have shown that free-living rhizospheric bacteria with ACC deaminase activity readily promote the nodulation process of several leguminous plants (Nascimento et al., 2016). These results indicate that the presence of bacteria with ACC deaminase activity can readily impact the colonization of other microorganisms present in the rhizosphere, including symbionts. Hence, under specific conditions bacteria with the ability to modulate plant ACC and ET levels may act as regulators of the plant microbiome. New studies are necessary to assess the specific role of bacteria with ACC and ET-modulation abilities in several aspects of the microbiome assembly (e.g., bacterial endophytism, aerial tissue colonization, microbiome composition).

### Strategies for the Creation of Inoculants with Increased Plant-Growth Promotion Abilities

Bacterial inoculants aiming to increase plant growth and development are the most promising alternatives to the use of potentially polluting agrochemicals. Since plants possess different ACC and ET production/sensitivity abilities, and, stress conditions readily increase plant ACC and ET levels, new strategies need to be considered to develop specific and efficient bacterial inoculants. These strategies need to be multidisciplinary and consider not only the added bacteria but also the plant host. In this sense, it is conceivable that ET and ACC insensitive plants will benefit less from the effects of ACC and ET-modulating bacteria. On the other hand, plants producing high levels of ACC and ET (naturally or induced by stress conditions) would certainly benefit from the presence of these bacteria. In fact, Chen et al. (2013) observed that the ACC deaminase-producing *Variovorax paradoxus* 5C2 promoted the growth of the *Arabidopsis* wild-type and the *Arabidopsis* ethylene-overproducing mutant *eto1-1* but not the ethylene-insensitive mutants, *etr1-1* and *ein2-1*, even though bacterial colonization of the root systems was similar. Furthermore, *V. paradoxus* 5C2 promoted the growth of *eto1-1* plants to a greater extent compared to all other treatments (Chen et al., 2013), indicating a positive feedback between plant ACC and ET production and the beneficial effect of the ACC deaminase-producing bacteria.

Finally, since ACC and ET inhibit the nodulation process, it is expected that rhizobial inoculants will benefit from the presence of free-living bacteria with ACC and ET modulation abilities. Hence, selecting ACC and ET-modulating rhizobia in concert with ACC and ET-modulating free-living bacteria may result in increased nodulation and leguminous plant growth.

## Author Contributions

All authors listed have made a substantial, direct and intellectual contribution to the work, and approved it for publication.

## Conflict of Interest Statement

The authors declare that the research was conducted in the absence of any commercial or financial relationships that could be construed as a potential conflict of interest.
