# Supplementary material for: Ethylene and 1-Aminocyclopropane-1-carboxylate (ACC) in Plant–Bacterial Interactions
Source: Front Plant Sci. 2018 Feb 22;9:114. doi: 10.3389/fpls.2018.00114 (PMC5827301; doi:10.3389/fpls.2018.00114)
Supplement: Supplementary file 1 [file Data_Sheet_1.docx]

**List of Abbreviations**

**ET-** Ethylene

**ACC-** 1-aminocyclopropane-1-carboxylate

**SAM-** S-adenosyl methionine

**MTA**- 5-methylthioadenosine

**ACS**- ACC synthase

**ACO-** ACC oxidase

**HCN**- Hydrogen cyanide

**M-ACC**- Malonyl-ACC

**G-ACC**- γ-glutamyl-ACC

**JA-ACC**- Jasmonoyl-ACC

**AMT-** ACC-N-malonyl transferase

**GGT**- γ-glutamyl-transpeptidase

**JAR1**- jasmonic acid resistance 1

**MPK**- Mitogen-ctivated Protein Kinase

**CDPK**- Calcium-dependent Protein Kinase

**LRRK-** Leucine-Rich Repeat Receptor Kinase

**ETR**- Ethylene Receptor

**ERS-** Ethylene Response Sensor

**EIN**- Ethylene Insensitive

**EIL**- Ethylene Insensitive-Like Protein

**ERF**- Ethylene Response Factor

**NFs**- Nodulation factors

**MAMPs**- Microbe-Associated Molecular Patterns

**FLG**- Flagellin

**EF-Tu**- Elongation factor-Tu

**PGN**- Peptidoglycan

**LPS**- Lipopolysaccharides

**NLPs**- Necrosis and Ethylene-inducing peptide 1 (Nep1)-Like Proteins

**DAMPs**- Damage-associated Molecular Patterns

**PRR**- Pattern Recognition Receptors

**PTI-** Pattern-Triggered Immunity

**ROS-** Reactive Oxygen Species

**HPRGs-** Hydroxyproline-Rich Glycoproteins

**FLS2-** Flagellin Sensitive 2

**FRK1-** Flg22-Induced Receptor-Like Kinase 1

**EFR**- EF-Tu Receptor

**LRR-RP**- Leucine-Rich Repeat Receptor Protein

**SOBIR1**- Suppressor of Brassinosteroid Insensitive 1 (BRI1)-Associated Kinase (BAK1)-interacting receptor kinase 1),

**LYM-** Lysin-motif Domain Proteins,

**CERK1**- Lysin-motif receptor kinase

**LORE-** Lectin S-domain-1 Receptor–Like Kinase

**PROPEP1**- Precursor of Peptide 1

**PEPR**- Pep1 Receptor Kinases

**BIK1-** Botrytis-Induced Kinase 1

**NB-LRR**- Nucleotide Binding and Leucine Rich Repeat Domains Proteins

**R**- Resistance Proteins

**ETI**- Effector-Triggered Immunity

**HR**- Hypersensitive Response

**ETO**- Ethylene-overproducing

**LYK-** Lysine Motif Domain-Containing Receptor-Like Kinase

**NFP**- Nodulation Factor Perception

**NFR1-** Nodulation Factor Receptor1

**RLK-** Receptor-Like Kinase

**MKK-** Mitogen-activated Protein Kinase Kinase

**MCP**- Methyl-Accepting Chemotaxis Protein

**RTX-** Rhizobitoxine

PHB- Poly-3-hydroxybutyrate

**AVG-** Aminoethoxyvinylglycine

**MVG-** Methoxyvinylglycine

**FVG-** Formylaminooxyvinylglycine

**PAs**- Polyamines

**EFE**- Ethylene -Forming Enzyme
